# Socioeconomic disparities associated with symptomatic Zika virus infections in pregnancy and congenital microcephaly: A spatiotemporal analysis from Goiânia, Brazil (2016 to 2020)

**DOI:** 10.1371/journal.pntd.0010457

**Published:** 2022-06-17

**Authors:** Luiza Emylce Pela Rosado, Erika Carvalho de Aquino, Elizabeth Bailey Brickley, Divania Dias da Silva França, Fluvia Pereira Amorim Silva, Vinicius Lemes da Silva, Angela Ferreira Lopes, Marilia Dalva Turchi

**Affiliations:** 1 Institute of Tropical Pathology and Public Health, Federal University of Goiás, Goiânia, Brazil; 2 Department of Obstetrics, of Maternal Children’s Hospital, Goiania, Brazil; 3 Department of Infectious Disease Epidemiology, London School of Hygiene & Tropical Medicine, London, United Kingdom; 4 Department of Surveillance, Health Secretary of Goiania, Goiania, Brazil; 5 Public Health Laboratory of Goias, Goiania, Brazil; 6 Faculty of Pharmacy, Federal University of Goias, Goiânia, Brazil; Universidade do Estado do Para: Universidade do Estado do Para, BRAZIL

## Abstract

The Zika virus (ZIKV) epidemic, which was followed by an unprecedented outbreak of congenital microcephaly, emerged in Brazil unevenly, with apparent pockets of susceptibility. The present study aimed to detect high-risk areas for ZIKV infection and microcephaly in Goiania, a large city of 1.5 million inhabitants in Central-West Brazil. Using geocoded surveillance data from the Brazilian Information System for Notifiable Diseases (SINAN) and from the Public Health Event Registry (RESP-microcefalia), we analyzed the spatiotemporal distribution and socioeconomic indicators of laboratory confirmed (RT-PCR and/or anti-ZIKV IgM ELISA) symptomatic ZIKV infections among pregnant women and clinically confirmed microcephaly in neonates, from 2016 to 2020. We investigated temporal patterns by estimating the risk of symptomatic maternal ZIKV infections and microcephaly per 1000 live births per month. We examined the spatial distribution of maternal ZIKV infections and microcephaly cases across the 63 subdistricts of Goiania by manually plotting the geographical coordinates. We used spatial scan statistics estimated by discrete Poisson models to detect high clusters of maternal ZIKV infection and microcephaly and compared the distributions by socioeconomic indicators measured at the subdistrict level. In total, 382 lab-confirmed cases of maternal ZIKV infections, and 31 cases of microcephaly were registered in the city of Goiania. More than 90% of maternal cases were reported between 2016 and 2017. The highest incidence of ZIKV cases among pregnant women occurred between February and April 2016. A similar pattern was observed in the following year, although with a lower number of cases, indicating seasonality for ZIKV infection, during the local rainy season. Most congenital microcephaly cases occurred with a time-lag of 6 to 7 months after the peak of maternal ZIKV infection. The highest estimated incidence of maternal ZIKV infections and microcephaly were 39.3 and 2.5 cases per 1000 livebirths, respectively. Districts with better socioeconomic indicators and with higher proportions of self-identified white inhabitants were associated with lower risks of maternal ZIKV infection. Overall, the findings indicate heterogeneity in the spatiotemporal patterns of maternal ZIKV infections and microcephaly, which were correlated with seasonality and included a high-risk geographic cluster. Our findings identified geographically and socio-economically underprivileged groups that would benefit from targeted interventions to reduce exposure to vector-borne infections.

## Author summary

The first wave of Zika virus (ZIKV) epidemic and its Congenital Zika Syndrome, has vanished. However, the consequences have remained for the affected children and families ever since.

In Brazil, the first cases of microcephaly, detected in the end of 2015 in the Northeast region, especially in coastal cities, quickly spread to other regions and cities in countryside of Brazil. Understanding the temporal and spatial dynamics of cases distribution is essential to identify areas of greater risk and enable preparedness for a future wave of cases.

In this study, we analyzed the spatiotemporal distribution of cases of ZIKV infection in pregnant women and cases of microcephaly in newborns by district, over a five-year period, in a large city in Midwest Brazil. Additionally, cases of microcephaly were correlated with the socioeconomic and structural conditions at the local level.

Our findings indicate heterogeneity in the spatiotemporal patterns of maternal ZIKV infections and microcephaly, which were correlated with seasonality and included a persistent high-risk geographic location (cluster) in the city of Goiania. We could identify geographically and socio-economically underprivileged groups, with higher risk for ZIKV infection, that would benefit from targeted interventions to reduce exposure to new vector borne infections.

## Background

The threat of Zika virus (ZIKV), which peaked between 2016 and 2017 and has been in decline over the subsequent four years, is probably not over [1]. Brazilian epidemiological surveillance services registered suspected cases and confirmed infections in 12 of the 27 Brazilian states in 2020 and in 3 states during the first months of 2021 [2,3], demonstrating that viral circulation persists, although at a smaller scale. It is hypothesized that a new outbreak in Brazil is feasible within a period of 6 to 10 years, due to the continued presence of competent mosquito vectors, the growing proportion of susceptible individuals, and the reported existence of ZIKV strains with higher transmissibility and pathogenicity [4].

Shortly after the first reported autochthonous transmission of ZIKV in the Northeast region (NE) of Brazil in April 2015[5,6] a large number of cases of a ‘dengue-like’ disease was reported throughout Brazil and many Latin American countries [7]. The rapid spreading of ZIKV was followed by an unexpected number of Guillain-Barré Syndrome and congenital microcephaly cases, initially reported in the NE region of Brazil [8,9]. From that region, the spatial distribution of ZIKV infection shifted to the Central-West and North regions, followed, as well, by an increase in microcephaly cases months after [10]. Surveillance data from the Brazilian Ministry of Health indicates that the majority of children with confirmed Congenital Zika Syndrome were born in late 2015 (n = 954 cases) and in 2016 (n = 1927 cases), with an abrupt decline in the following years. In 2019, only 55 cases were confirmed in Brazil [11].

Robust evidence now confirms the role of congenital ZIKV infection in the pathogenesis of microcephaly and other malformations, together recognized as Congenital Zika Syndrome (CZS) [12–14]. Yet, the magnitude of the ZIKV epidemic and the subsequent outbreak of microcephaly have not been homogeneous across geographic regions and over time. Several studies using national and international data have reported significant geographic variations in the burden of ZIKV infection and CZS across countries and macro regions [15–22]. At the more local level, there are still gaps in understanding concerning environmental and socioeconomic factors that are associated with ZIKV infection and CZS risks. In this study, we aimed to analyze the temporal and spatial distributions of symptomatic ZIKV infections among pregnant women and the occurrence of microcephaly among live births in a large city, located in the Central-West part of Brazil, and their relation to socioeconomic parameters.

## Methods

### Study design and data sources

**Fig 1:**
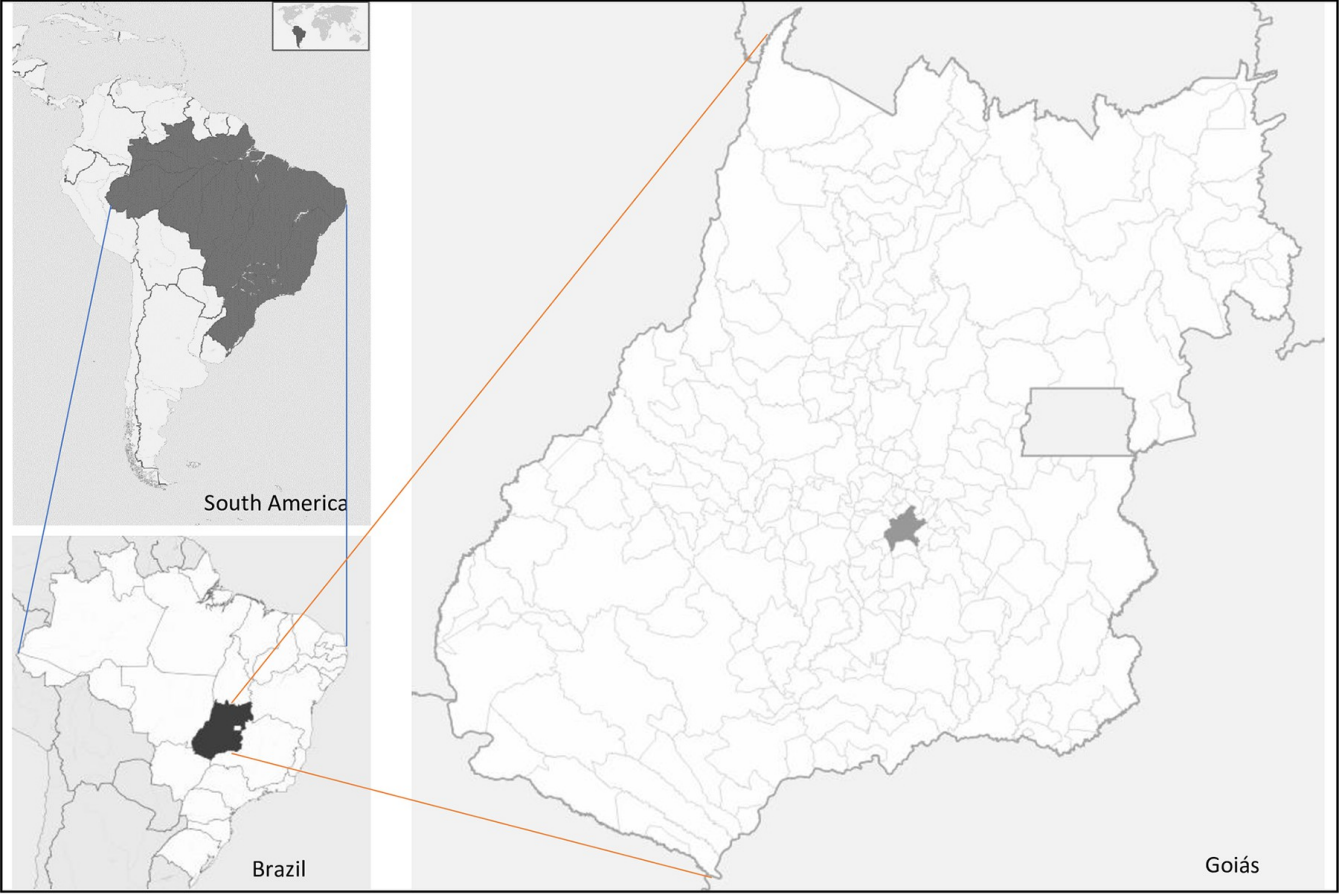
Location map of the State of Goias and the city of Goiania in Brazil, Brazilian Institute of Geography and Statistics (IBGE), 2022 https://portaldemapas.ibge.gov.br/portal.php#homepage).

This is a retrospective ecological study conducted in the city of Goiania, which is the capital of the state of Goiás. Goiania is located in the Central-West region of Brazil (Latitude, longitude: -16.68, -49.26) and has an estimated population of 1.5 million inhabitants residing across 63 subdistricts [23]. In 2010, its Human Development Index (HDI) was 0.799, and the Gini Index (GI), that measures the income distribution across a population, was 0.59 [23], indicating a high degree of inequality (Fig 1).

The data for these analyses were obtained from the Brazilian Information System for Notifiable Diseases (SINAN) and from the Public Health Event Registry for Microcephaly system (RESP-microcefalia). Since 2016, ZIKV infection has been a compulsory notifiable disease in Brazil [24], and the SINAN platform was used to identify cases of symptomatic pregnant women, with laboratory confirmed ZIKV infection, in Goiania, from 1 January 2016 to 31 October 2020. ZIKV infections were laboratory confirmed using reverse transcription-polymerase chain reaction (RT-PCR) testing of serum collected within 5 days of symptom onset or urine samples collected within 10 days and/or anti-ZIKV immunoglobulin (Ig) M enzyme-linked immunosorbent assay (ELISA) testing of serum. All laboratory tests were routinely performed at the Dr. Giovanni Cysneiros State Laboratory of Public Health (LACEN, Goiás, Brazil). Data on microcephaly were retrieved from the official RESP-microcefalia database. Microcephaly cases were defined according to operational definitions from the Brazilian Ministry of Health as a head circumference of two or more standard deviations below the mean for age and sex using the INTERGROWTH standard curves [12].

The temporal and spatial patterns were investigated as the frequency of laboratory- confirmed maternal ZIKV infections and microcephaly cases among live births by month and subdistrict. To estimate the denominators, data on the number of newborns were first obtained from the National Information System on Live Births (SINASC) through the website of the Department of Informatics of the Unified Health System [13]. These data were then projected proportionally to the number of women of childbearing age in the subdistricts of Goiânia, according to the 2010 census, and used as a proxy for the number of live births in each of them.

Data on the socioeconomic indicators of sanitation, household income, and self-identified race/ethnicity were obtained from the Brazilian Institute of Geography and Statistics (IBGE) [23]. The census subdistricts were categorized in quartiles based on the: (i) percentage of households with inadequate sanitation (i.e., septic tank, other means of sewage disposal, and no disposal), (ii) percentage of households with nominal per capita household income of up to half of the minimum wage for the Brazilian population, and (iii) percentage of residents self-identifying as white.

### Statistical analysis

For the analysis of seasonality, cases of ZIKV infection and microcephaly were plotted in bar charts by month for 2016 and 2017. The Kruskal Wallis test was used to investigate the presence of seasonality in this time series [6]. The crude relative risks of symptomatic maternal ZIKV infection and microcephaly were estimated to compare the different years; *p* values were obtained from chi-squared tests. Owing to the low number of cases within given months, data have been aggregated by quarters (3-month blocks) to make the findings more easily interpretable.

The spatial distribution of ZIKV-infected pregnant women and microcephaly cases were investigated using scan statistics to identify clusters of high-risk sub-districts from 2016 to 2017 and in relation to sociodemographic variables. The cases were manually georeferenced using the Google Earth program by household address. The incidence of Zika in pregnant women and the prevalence of microcephaly were aggregated according to the sub-district of residence. To detect clusters of high-risk subdistricts, Kulldorff’s purely spatial scan statistic was applied considering the population at risk per area. In this method, a circular window is established on the map, centering consecutively on predefined coordinates (centroids) within the study region. An infinite number of distinct geographic circles was created, and then submitted to statistical analysis to define it as a high-risk cluster [25–29]. For this study, the geographic centers of the 63 sub-districts of Goiania were considered as the centroids, in which data about diagnosis and number of newborns were geographically allocated. The subdistrict boundary polygons were used only for presentation of clusters on the map. Over each centroid, the radius of the circular window varied continuously in size from zero to a previously established upper limit, with each window including different sets of neighboring sub-districts.

Two criteria of inclusion were defined by the authors as a parameter for the maximum size of a cluster: a radius of 5 kilometers and 50% of the population at risk of ZIKV infection and its complications [26,28]. To detect the spatial location of the clusters, and to evaluate their statistical significance, the log likelihood ratio test statistic (LRTS) based on the discrete Poisson distribution was used [25,29]:

$$LTRS=máx{\left(\frac{Yin}{Ein}\right)}^{Yin}{\left(\frac{Yout}{Eout}\right)}^{Yout}$$

where *Yin* is the number of cases detected in the established region defined by circle of interest, and *Ein* is the expected number expected under the null hypothesis. *Yout* and *Eout* are detected and expected number of cases out of each region, respectively.

For each centroid and size of the scan window, the alternative hypothesis was that there was a high-risk inside the window as compared to outside. Using the Monte Carlo simulation, independent data sets were generated under the null hypothesis, and the empirical distribution of the LRTS was calculated to evaluate the significance of the statistics of all possible groupings. Geographically overlapping clusters were not reported [26,28].

The SaTScan program version 9.1.111–12[30] was used for the spatial analysis with the purpose of identifying clusters of risk. To create thematic maps, the Google Earth program [31] was used. The spatial distribution was presented per reporting year (2016 and 2017) and Terra View 4.2.2 software [32] was used to elaborate the maps.

All other statistical analyses were performed in Stata 14.0 (StataCorp. 2015. *Stata Statistical Software*: *Release 14*. College Station, TX: StataCorp LP), the level of significance was set at p < .05 for all statistical tests, and the 95% confidence interval (95% CI) was calculated.

## Results

In this ecological study done in Goiania, Brazil, we identified a total of 382 laboratory confirmed symptomatic maternal ZIKV infections and 31 cases of congenital microcephaly, reported to Brazilian surveillances systems (SINAN and RESP). Almost all cases of confirmed ZIKV infection in pregnant women (378/382, 99.0%) were reported during the 2016 and 2017 period, during which there was a crude rate of 9.2 cases/1000 live births (95% CI 8.3–10.2). Four cases of maternal ZIKV infection and five cases of microcephaly were registered from 2018 to 2020. Since there were very few cases after the 2016–2017 epidemic, they were not included in the subsequent analyses presented in Table 1.

**Table 1:** Zika virus infection (ZIKVi) in pregnant women, and microcephaly grouped according to a period of 3 months, and a year of notification / birth in Goiania.

		Confirmed Zika cases in pregnant women	Cases of CMC	Live births	Zika in pregnancy incidence*	CI 95 %	Relative risk for Zika in pregnancy	CI 95 %	P- value	CMC prevalence*	CI 95 %	CMC prevalence ratio	CI 95 %	P-value
** **	**Goiânia (total)**	378	26	41138	9.2	(8.3; 10.2)	----------			0.4	(0.2; 0.6)			
**Years**	**2016**	335	24	21125	15.9	(14.2; 17.6)	**7.6**	**(4.8; 12.8)**	**<0.001**	1.1	(0.7; 1.6)	**11.4**	**(1.6; 473.5)**	**0.003**
	**2017**	43	2	20013	2.1	(1.6; 2.8)	1	----	----	0.1	(0.0; 0.3)	1	----	----
**2016**	**Jan/Feb/Mar**	219	1	5573	39.3	(34.4; 44.6)	**49.1**	**(24.6; 114.5)**	**<0.001**	0.2	(0.0; 0.8)	**0.1**	**(0.0; 0.3)**	**<0.001**
	**Apr/May/Jun**	107	1	5670	18.9	(15.5; 22.6)	**23.6**	**(11.7; 55.5)**	**<0.001**	0.2	(0.0; 0.8)	**0.1**	**(0.0; 0.3)**	**<0.001**
	**Jul/Aug/Sep**	5	10	5110	1	(0.3; 2.1)	1.3	(0.4; 3.6)	0.648	2	(0.9; 3.5)	0.8	(0.4; 1.5)	0.461
	**Oct/Nov/Dec**	4	12	4772	0.8	(0.2; 2.0)	1	----	----	2.5	(1.3; 4.2)	1	----	----
**2017**	**Jan/Feb/Mar**	14	0	5280	2.7	(1.5; 4.3)	**13.5**	**(3.4; 117.1)**	**<0.001**	0	(0.0; 0.5)	0	(0.0; 5.3)	0.25
	**Apr/May/Jun**	28	1	5371	5.2	(3.5; 7.4)	**26.0**	**(6.8; 220.3)**	**<0.001**	0.2	(0.0; 0.9)	**0.9**	**(0.1; 13.8)**	**1.000**
	**Jul/Aug/Sep**	1	1	4841	0.2	(0.0; 1.0)	1	----	----	0.2	(0.0; 1.0)	1	----	----
	**Oct/Nov/Dec**	0	0	4521	0	(0.0; 0.6)	0	(0.0; 5.32)	0.25	0	(0.0; 0.6)	0	(0.0; 5.3)	0.25
**Total**	**Jan/Feb/Mar**	233	1	10853	21.5	(16.3; 22.9)	**49.9**	**(9.4; 50.6)**	**<0.001**	0.1	(0.0; 0.3)	**0.1**	**(0.0; 0.9)**	**0.022**
	**Apr/May/Jun**	135	2	11041	12.2	(9.7; 13.3)	**28.4**	**(5.3; 32.3)**	**<0.001**	0.2	(0.0; 0.4)	**0.1**	**(0.0; 0.9)**	**0.022**
	**Jul/Aug/Sep**	6	11	9951	0.6	(0.1; 0.8)	1.4	(0.1; 2.2)	0.388	1.1	(0.3; 1.2)	0.9	(0.3; 2.8)	0.804
	**Oct/Nov/Dec**	4	12	9293	0.4	(0.1; 1.1)	1	----	----	1.3	(0.4; 1.3)	1	----	----

* Prevalence and incidence calculated by 1000 live birth. CMC = Congenital microcefaly

A sharp reduction was observed between the years of 2016 and 2017 in the incidence of ZIKV infections in pregnant women per 1000 live births, which dropped from 15.9 (95% CI 14.2–17.6) to 2.1 (95% CI 1.6–2.8), with a relative risk of ZIKV infection of 7.6 (95% CI 4.8–12.8) (p <0.001) comparing 2016 versus 2017. In parallel, the prevalence of congenital microcephaly was (1.1 95% CI 0.7–1.6) and 0.1 (95% CI 0.0–0.3) per 1000 live births, respectively in those years, showing a prevalence ratio of microcephaly of 11.4 (95% CI 1.6–473.5) comparing 2016 versus 2017 (Table 1).

Looking at the distribution of cases by trimester of the year, the highest incidence of ZIKV infection peaked in the first three months of 2016 (39.3/1000 live births; 95% CI 34.4–44.6), and the peak prevalence of microcephaly was observed in the last three months of the same year (2.5/1000 live births; 95% CI 1.3–4.2).

**Fig 2:**
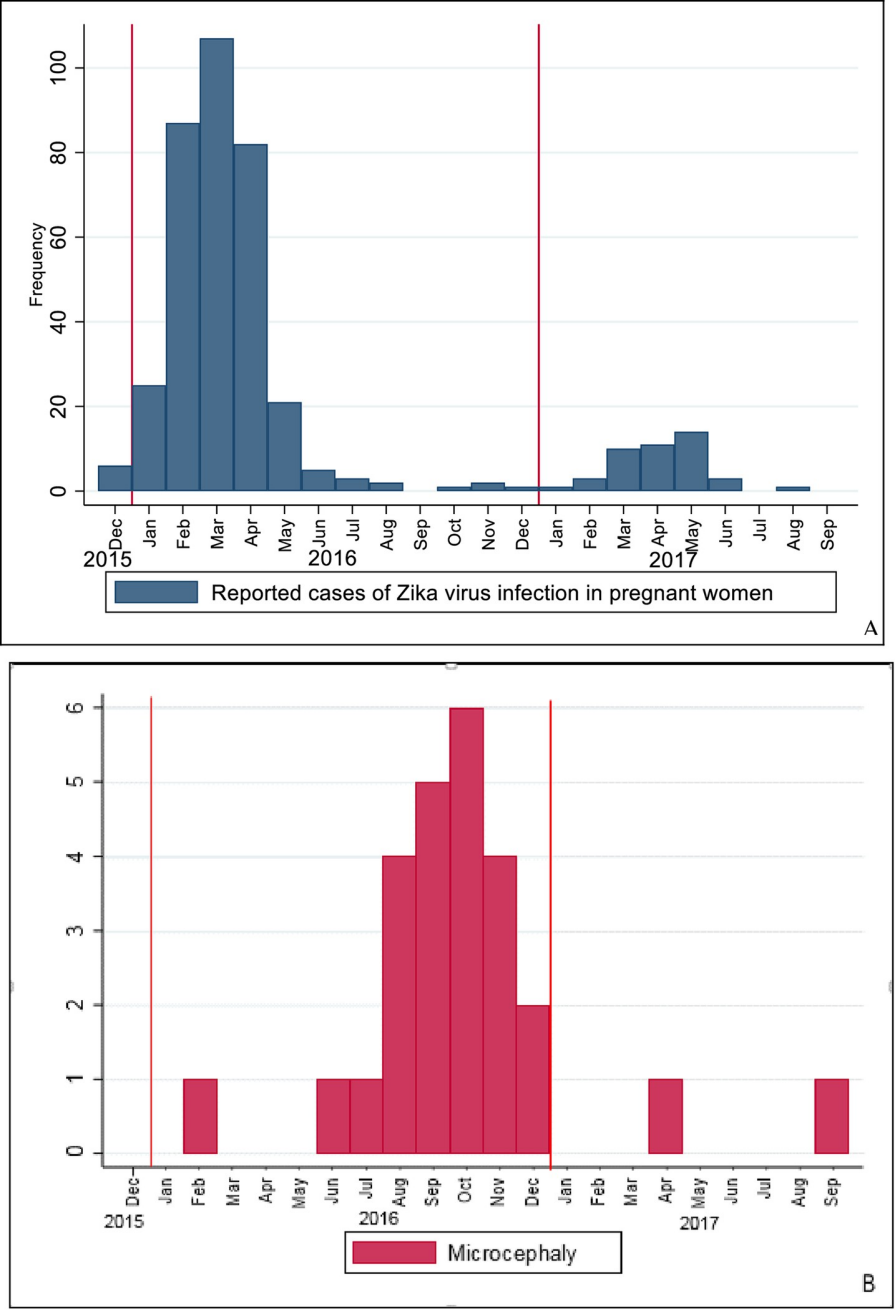
A- Confirmed cases of Zika virus infection (ZIKVi) in pregnant women, and cases of microcephaly according to month and year of notification, and birth date in the city of Goiania (2015 to 2017); B- Cases of microcephaly according to month and year of notification, and birth date in the city of Goiania (2016 to 2017). The vertical red lines indicate changes in the calendar year.

The greatest absolute incidence of confirmed ZIKV infection among pregnant women occurred between February and April 2016, with a significant decrease in subsequent months. A similar pattern was observed in the following year, from March to May, although with a much lower number of cases (Fig 2A), confirming a pattern of seasonality of ZIKV cases, with higher incidence during the local rainy season, which runs from late October to early April and peaks from November to March (p <0.001). Most cases of congenital microcephaly were reported from September to October 2016, with a time-lag of 6 to 7 months after the peak of ZIKV infection among pregnant women (Fig 2B), demonstrating a temporal relationship with ZIKV infection in pregnant women.

**Fig 3:**
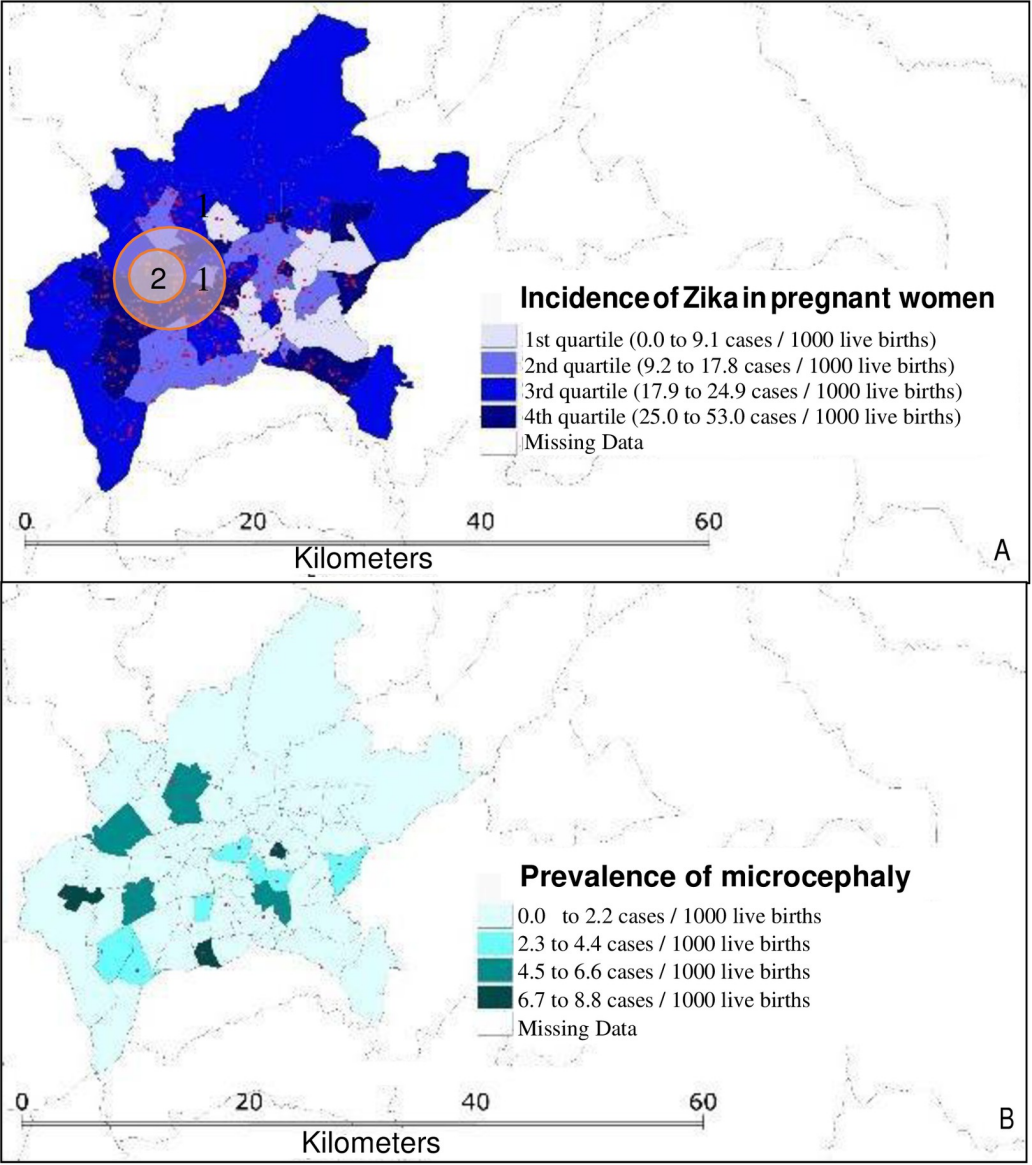
A- Cases and quartiles of incidence of Zika virus infection (ZIKVi) in pregnant women according to the census sub-district of the city of Goiania (2016/2017). B- Cases and quartiles of prevalence of microcephaly according to the census sub-district of the city of Goiania (2016/2017). 1 –Spatial cluster of high risk of ZIKVi in pregnant women, 2016. 2 –Spatial cluster of high risk of ZIKVi in pregnant women, 2017. https://ibge.gov.br/geociencias/organizacao-do-territorio/estrutura-territorial/15774-malhas.html.

Laboratory-confirmed ZIKV cases among pregnant women were reported all over the city, with 85.7% of the sub-districts having at least one case per 1000 live births in 2016 and 2017. Fig 3 shows the spatial distribution of the incidence of symptomatic ZIKV in pregnant women (3-A) and the prevalence of microcephaly throughout the sub-districts of Goiania (3-B). The highest incidence of ZIKV in pregnant women was observed in the southwest region of the municipality in both 2016 and 2017. Despite having the same geographical location in both years, the size of the geographic cluster was bigger in 2016. There was also a wide distribution of microcephaly cases across the sub-districts of Goiania, although we were unable to detect a single high-risk cluster.

The incidence and the relative risk of symptomatic maternal ZIKV infection and the prevalence of congenital microcephaly per 1000 live births, according to socioeconomic variables, in quartiles, are shown in Table 2. The relative risk of ZIKV infections among pregnant women was higher in subdistricts with a higher percentage of households with inadequate sanitation and with nominal income per capita up to ½ minimum wage. The relative risk of ZIKV infection among pregnant women was also lower in areas with higher percentages of residents identifying as white. Looking at microcephaly prevalence, we observed no statistically significant differences across quartiles of sanitation or income. Similarly, we observed no overall gradient in the risks of microcephaly in relation to the percentage of self-identified white residents; however, risks were lower in subdistricts in the third quartile compared to those in the first (Table 2).

**Table 2:** Zika virus infection (ZKVI) in pregnant women, and microcephaly according to socioeconomic variables in Goiania (2016/2017).

		Confirmed Zika cases in pregnant women	Cases of CMC	Live births (average)	Zika in pregnant women incidence*	CI 95 %	Relative risk Zika in pregnant women incidence	CI 95 %	P-value	CMC prevalence*	CI 95 %	CMC prevalence ratio	CI 95 %	P-value
**Percentage of households with inadequate sanitary sewage**	Q1 (0.0 to 2.0%)	69	8	5487	12.6	(9.8; 15.8)	1	----	----	1.5	(0.7; 2.8)	1	----	----
	Q2 (2.1 to 9.0%)	97	3	4612	21	(17.2; 25.5)	**1.7**	**(1.3; 2.1)**	**<0.001**	0.7	(0.1; 1.7)	0.4	(0.2; 1.2)	0.093
	Q3 (9.1 to 43.1%)	82	3	4914	16.7	(13.7; 20.5)	**1.3**	**(1.0; 1.7)**	**0.017**	0.6	(0.2; 1.7)	0.4	(0.1; 1.1)	0.053
	Q4 (43.2 to 99.8%)	130	12	6322	20.6	(17.3; 24.3)	**1.6**	**(1.3; 2.5)**	**<0.001**	1.9	(1.0; 3.2)	1.3	(0.6; 2.7)	0.5
**Percentage of households with nominal monthly household income per capita up to ½ minimum wage**	Q1 (2.4 to 8.9%)	37	4	3399	10.9	(7.8; 14.9)	1	----	----	1.2	(0.4; 2.8)	1	----	----
	Q2 (9.0 to 11.9%)	107	5	6050	17.7	(14.6; 21.2)	**1.6**	**(1.3; 2.1)**	**<0.001**	0.8	(0.3; 1.8)	0.7	(0.2; 1.8)	0.383
	Q3 (12.0 to 19.0%)	112	6	5556	20.2	(16.7; 24.1)	**1.9**	**(1.5; 2.4)**	**<0.001**	1.1	(0.4;2.2)	0.9	(0.4; 2.3)	0.838
	Q4 (19.1 to 43.5%)	122	11	6329	19.3	(16.1; 22.9)	**1.8**	**(1.4; 2.3)**	**<0.001**	1.7	(0.9; 3.0)	1.5	(0.6; 3.2)	0.362
**Percentage of white residents (self-declared)**	Q1 (28.4 to 40.9%)	115	11	6427	17.9	(14.9; 21.4)	1	----	----	1.7	(0.9; 2.9)	1	----	----
	Q2 (41.0 to 47.6%)	112	5	5272	21.2	(17.6; 25.4)	1.2	(0.9; 1.4)	0.095	0.9	(0.3; 2.1)	0.6	(0.2; 1.3)	0.122
	Q3 (47.7 to 56.7%)	107	4	5723	18.7	(15.4; 22.5)	1	(0.8; 1.3)	0.676	0.7	(0.2; 1.7)	**0.4**	**(0.1; 1.0)**	**0.043**
	Q4 (56.8 to 81.7%)	44	6	3913	11.2	(8.3; 14.9)	**0.6**	**(0.5; 0.8)**	**<0.001**	1.5	(0.6; 3.2)	0.9	(0.4; 1.9)	0.728

** Prevalence and incidence calculated by 1000 live birth. CMC = Neonatal microcefaly

**Fig 4:**
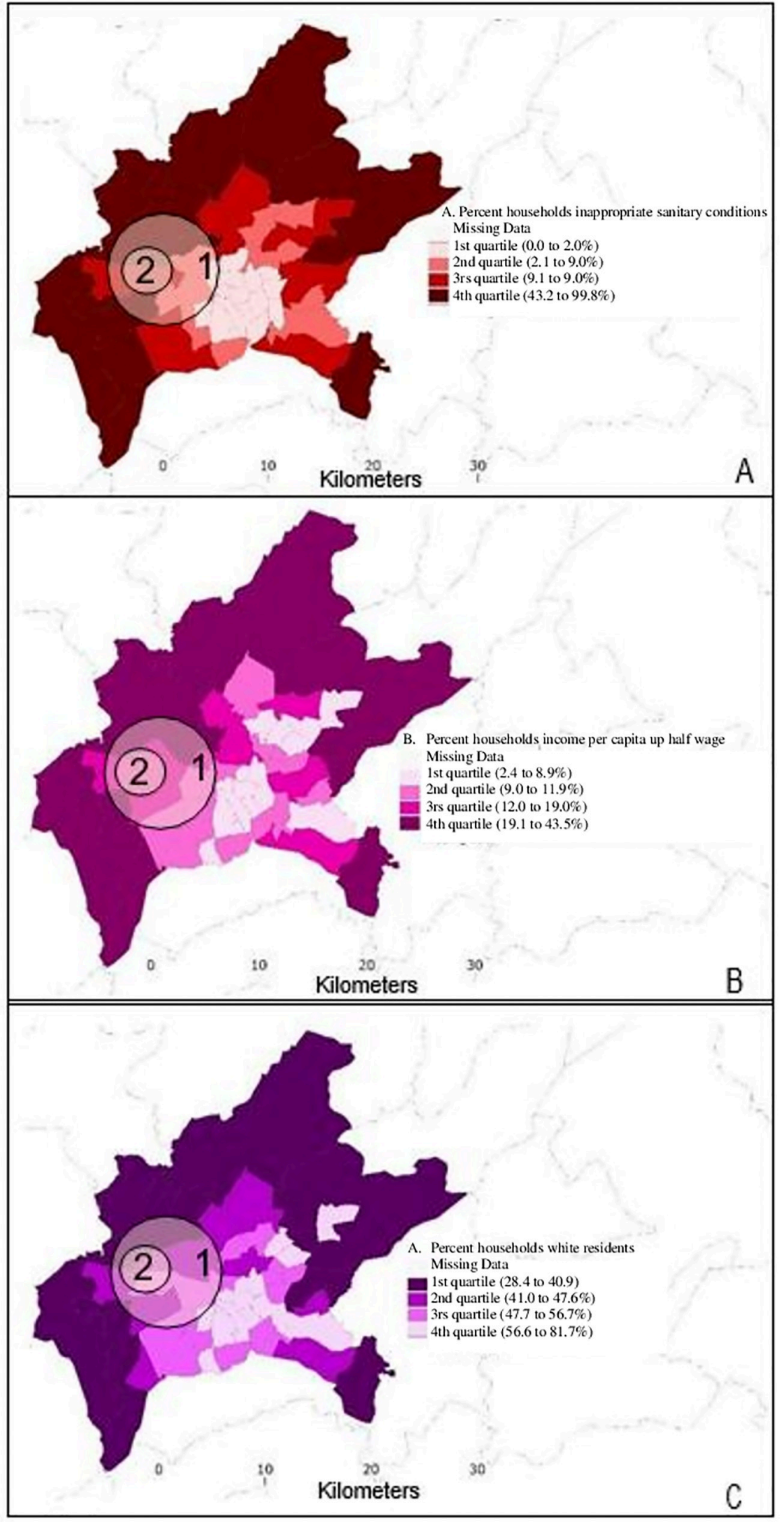
Sociodemographic conditions of sub-district of Goiania, by the census of 2010: A- Quartiles of percentage of households with inappropriate sanitary conditions (e.g, septic tank, other means of exhaustion, and no depletion). B- Quartiles of percentage of households with nominal monthly household income per capita up to a half of minimum wage. C- Quartiles of percentage of self-declared white residents. 1 –Spatial cluster of high risk Zika virus infection (ZIKVi) in pregnant women, 2016. 2 –Spatial cluster of high risk ZIKVi in pregnant women, 2017. https://ibge.gov.br/geociencias/organizacao-do-territorio/estrutura-territorial/15774-malhas.html.

Fig 4 shows the sociodemographic conditions of the subdistricts of Goiania, by the census of 2010, and its relation to the high-risk cluster of ZIKV infections in 2016 and 2017, demonstrating that they overlapped with areas of intermediate socioeconomic conditions.

## Discussion

This ecological study investigated the temporal and spatial distribution of confirmed maternal ZIKV infections and microcephaly cases in relation to socioeconomic variables in a large city in Brazil over a five-year period. Most cases occurred during 2016 and 2017; however, despite of the end of the outbreak, sporadic cases were still notified in subsequent years, indicating continued ZIKV circulation in the city. Our data showed a high incidence of ZIKV infection during the local rainy season, with a higher-risk cluster identified in the same geographical area over two consecutive years. These findings reinforce the need for better local vector control and for individually targeted protective measures, especially in that location, for pregnant women.

Consistent with previous ecological studies [33], this investigation found that living in areas with better socioeconomic indicators (i.e., improved sanitation and income) and with a higher percentage of self-identified white residents was associated with lower risks of ZIKV infections in pregnancy. The association between socioeconomic indicators and microcephaly aligns with a previous study from the city of Recife, in Northeast Brazil [34], which found a strong association between the prevalence of microcephaly during the 2015–2016 epidemic and poor living conditions defined by the percentage of household heads with an income below two times minimum wage. Further, the observed reduced risk of ZIKV infections in areas with a higher percentage of white individuals is likely because, in the setting of Brazil, race/ethnicity is associated with socioeconomic status. Black (*Preto*) and brown (*Pardo*) individuals may be subject to structural racism and are more likely to have less access to education and health services, to live in areas with worse environmental conditions, and to be more exposed to vector-borne infections than their white counterparts [35].

Inadequate water treatment as well as precarious sanitary sewage have been previously related to vector-borne infections [33]. Those unsatisfactory environmental conditions become more obvious soon after the start of the rainy season, particularly in tropical and subtropical areas, where the prevalence of water puddles can facilitate breeding sites for *Aedes spp*. mosquitoes, which has been observed, not only in our study but, in other Brazilian states [36] and South American and Caribbean countries [37].

Correlations between ZIKV infections and socioeconomic disparities in ecological studies can be, sometimes, contradictory [38]. In the Northern Brazilian state of Rio Grande do Norte, it was found that ZIKV infections in 2015–2016 had higher Average Incidence Rate (AIR) in municipalities with better median income as well as higher notification rates of violence, likely reflecting improved surveillance and local organization of public health services [39]. Another study done in Espirito Santo, Southern Brazil, that analyzed data from 2016, no social determinants were found to be associated with ZIKV infections neither in the general nor pregnant populations, but in this site, the socioeconomic indicators were much higher overall than the national Brazilian averages. Similarly, analyses of arbovirus infections in two Latin American cities [38], Fortaleza in Brazil and Medellin in Colombia, from the period of 2007 to 2017, found almost no correlation between ZIKV infections and inequalities, although the incidence of ZIKV infections were much lower than for Dengue and Chikungunya in those locations.

As seen in previous studies [10,16,40,41], our investigation revealed that the microcephaly cases were concentrated in the same areas with higher incidences of ZIKV in pregnant women, and the peak of microcephaly cases occurred six to seven months after the highest incidence of ZIKV infections. It is known that relationship between time and space are important features to confirm causality between an insult and a possible disease [14], and this study corroborates the evidence of an association between ZIKV infection and microcephaly.

Of note, the high-risk clusters of ZIKV infection overlapped in 2016 and 2017, suggesting that an important focal point of transmission was maintained in that region of the city. It is noteworthy that, even though there is a local effort to control potential breeding sites of mosquitoes [42], that region is known to have many junkyards and car wrecks that might contribute to vector nurseries as there are lots of spots that can accumulate standing water and facilitate larval growth.

Overall, the number of reported ZIKV cases in Brazil decreased from 216.207 cases in 2016 to 17.338 in 2017, with population immunity thought to be the main cause of the decline [3]. Similarly, in our study, ZIKV infection decreased in cases and numbers of affected sub-districts in 2017 and, subsequently, in the following years, likely representing a reduced circulation of ZIKV in the general population [43]. Even considering the possibility of underreporting, especially after the first year of the epidemic, it is important to remind that during 2017, only 11.1% of the notifications for suspected ZIKV cases in Brazil were later laboratory-confirmed, against 88.8% in the previous year, as well as observed in the city of Goiania (12.5% in 2017 vs. 75.5% in 2016), reinforcing the hypothesis of lower viral circulation [44].

It is critical to emphasize that the serodiagnosis of ZIKV infection in Brazil has been hindered by the cross-reactivity among flavivirus antibodies and by the fact that dengue has been endemic in Brazil for more than 30 years [45,46]. The unknown rate of asymptomatic ZIKV cases in Brazil makes it difficult to ascertain true population-level exposure, although serosurvey conducted in Salvador in Northeast (NE) Brazil suggested a peak seroprevalence of 63% by 2016[47,48]. In line with that finding, study performed with mothers that gave birth during the 2015–16 outbreak in the city of Recife NE Brazil found anti-ZIKV IGG positive in 61,3% of them, suggesting a high rate of seropositivity in that region.

This georeferenced analysis of ZIKV infections and microcephaly cases, obtained from the official notification data system, allowed the identification of priority areas for surveillance and vector control. Specifically, our findings highlight socioeconomic disparities in the geographic distribution of ZIKV infections among pregnant women. It may also assist as a reference for future studies about the dispersion of ZIKV infection and other vector-borne diseases in the city of Goiania [28,29,43].

However, our findings are susceptible to limitations that are inherit to ecological studies, namely that the findings describe an association at the population rather than individual level and that there may be important sources of residual confounding [49]. In addition, we note that there could be misclassification in our exposure variables as socioeconomic conditions were based on the last census data in Brazil, which was conducted in 2010. While local conditions may have changed in the six years preceding the beginning of this study period, these census data are the most recent official data available and have been used in a series of similar ecological studies examining socioeconomic disparities in the distribution of ZIKV cases [23].

An additional limitation is the use of secondary data and its potential for underreporting, especially of those patients who attended private health clinics, which are prone to lower reporting of notifiable diseases than public clinics [38]. Although the notification of ZIKV cases in pregnant women became mandatory in Brazil as of February 2016[50], it is difficult to ascertain the potential for underreporting of symptomatic ZIKV infections in pregnant women in Goiania included in this analysis. Nonetheless, during the first year of the Brazilian epidemic, the confirmatory tests by RT-PCR for ZIKV were performed mainly in the public reference laboratories, and so it is estimated that most known symptomatic cases of ZIKV in pregnant women, in Goiania, would be identified and included in the present study. An additional limitation could arise due to inaccuracies in the geocoding of cases’ home addresses. For this study, we elected to use Google Earth, which has been previously shown in a study in Portugal to have 90% accuracy and less positional error than other geocoding tools [51], although we acknowledge that other software (e.g., ArcGIS, QGIS) may have offered advantages with respect to geographic coverage and processing time [52].

Another limitation was that information on the number of pregnant women per sub-district were not available, so the number of newborns per sub-district was used as a proxy marker. It is important to note that the number of newborns is smaller than the number of pregnant women, since it does not include abortions and stillbirths. However, it is likely a good approximation of the number of pregnant women in the municipality.

## Conclusions

Our findings indicate heterogeneity in the spatiotemporal patterns of maternal ZIKV infections and microcephaly, which were correlated with seasonality and included a high-risk geographic cluster in the city of Goiania.

We identified geographically and socio-economically underprivileged groups that would benefit from targeted interventions to reduce exposure to vector-borne infections.
